# Spinal arachnoid diverticula in cats: Clinical presentation, diagnostic imaging findings, treatment, and outcome

**DOI:** 10.1111/jvim.17294

**Published:** 2024-12-30

**Authors:** João Miguel De Frias, Sofie F. M. Bhatti, George Nye, Rita Gonçalves, Tom Harcourt‐Brown, Angela Fadda, Katia Marioni‐Henry, Hannah Padley, Steven De Decker

**Affiliations:** ^1^ Department of Clinical Science and Services Royal Veterinary College, University of London London UK; ^2^ The Royal (Dick) School of Veterinary Studies Hospital for Small Animals University of Edinburgh Midlothian UK; ^3^ Small Animal Department, Faculty of Veterinary Medicine Ghent University Ghent Belgium; ^4^ Highcroft Veterinary Referrals CVS Group Bristol UK; ^5^ Small Animal Teaching Hospital University of Liverpool Liverpool UK; ^6^ Langford Vets University of Bristol Bristol UK; ^7^ The Queen's Veterinary School Hospital University of Cambridge Cambridge UK

**Keywords:** arachnoid cyst, feline, spinal cyst, subarachnoid diverticulum

## Abstract

**Background:**

Spinal arachnoid diverticulum (SAD) is considered a rare disease in cats. Previous reports mainly classified SAD in cats as acquired.

**Hypothesis/Objectives:**

The aim of this study was to describe the signalment, clinical presentation, diagnostic imaging findings, and outcome in a group of cats with SAD.

**Animals:**

Twenty‐one client‐owned cats.

**Methods:**

Multicenter observational retrospective review of the medical records of cats diagnosed with SAD by magnetic resonance imaging.

**Results:**

Most cats were Domestic Short Hair (67%), male (63%), and had a wide range of ages (18 weeks to 13 years old). Neuroanatomical localization was consistent with a T3‐L3 myelopathy in 18 cats (86%) and C1‐C5 myelopathy in 3 cats (14%). One cat with a C1‐C5 myelopathy demonstrated bilateral vestibular clinical signs. One cat (5%) had fecal incontinence. Most cats demonstrated a chronic, progressive, nonlateralized, nonpainful myelopathy. No underlying previous or concurrent spinal condition was found in 48% of the cats. No difference in age, body weight, breed, sex, treatment, or outcome was found between cats with or without a concurrent spinal disorder. One cat was euthanized after diagnosis. Six cats improved, 1 deteriorated and 1 remained static after surgery, whereas 3 cats improved, 5 deteriorated and 4 remained static after medical management on the short‐term outcome. Overall, 73% (8/11) of the cats deteriorated on available long‐term follow‐up information.

**Conclusions and Clinical Importance:**

Spinal arachnoid diverticulum should be considered for cats with chronic, progressive, symmetrical, nonpainful myelopathy, particularly if male and with a history of spinal disease or surgery.

**Abbreviations**
BALT GRAD: T2‐weighted thin‐slice gradient echo sequence
CSF: cerebrospinal fluid
FGS: feline grimace scale
FIP: feline infectious peritonitis
FLAIR: fluid‐attenuated inversion recovery
FMPI: feline musculoskeletal pain index
HASTE: half‐Fourier acquisition single‐shot turbo spin‐echo pulse sequence
MRI: magnetic resonance imaging
SAD: spinal arachnoid diverticula
SM: syringomyelia
T2W: T2‐weighted
T1W: T1‐weighted

## 1 INTRODUCTION

Spinal arachnoid diverticulum (SAD) is characterized as a focal dilatation of the subarachnoid space that causes progressive spinal cord compression and subsequent clinical signs.^1^, ^2^, ^3^ In cats, SAD is considered rare, with literature limited to case reports.^4^, ^5^, ^6^, ^7^, ^8^, ^9^, ^10^, ^11^, ^12^ Cats with SAD have so far been exclusively classified as acquired, suspected to have developed after another disease process, including diagnoses of vertebral malformation, acute noncompressive nucleus pulposus extrusion, vertebral fracture, feline infectious peritonitis (FIP) myelitis, and vertebral instability because of congenital hypothyroidism.^4^, ^5^, ^6^, ^7^, ^8^, ^9^, ^10^, ^11^ Therefore, this secondary nature suggests that SAD formation in cats is a sequela of disease or trauma. Nevertheless, a report of a 7‐year‐old male neutered Ragdoll, with a recurrence of SAD after surgery, did not specify if an underlying cause for SAD formation was identified when first presented.^12^

In contrast, SAD in dogs can be divided into acquired or primary forms in dogs although its pathophysiology is not completely understood.^1^ Spinal arachnoid diverticula in dogs is well‐characterized, with specific breed predispositions, distinctive clinical characteristics, and predictable outcomes after medical and surgical treatment.^1^, ^2^, ^3^, ^13^

Given the current paucity of literature on the subject, it remains challenging to identify clinical characteristics that could be suggestive of SAD in cats or to predict the outcome after medical or surgical treatment. The aim of this study is, therefore, to describe the signalment, clinical presentation, diagnostic imaging findings, and outcome in a larger cohort of cats with SAD. We hypothesize that SAD in cats exhibits analogous classification (primary and acquired) and semiology to those observed in canine SAD.

## 2 MATERIALS AND METHODS

Ethics approval was granted by the Social Sciences Research Ethical Review Board (SSRERB) at the Royal Veterinary College (RVC) (reference number URN SR2021‐0149).

Medical records of cats diagnosed with SAD from 6 institutions in the United Kingdom and 1 in Belgium were retrospectively reviewed: 6 teaching veterinary hospitals, Queen Mother Hospital for Animals (Royal Veterinary College, University of London), Small Animal Teaching Hospital (University of Liverpool), Langford Vets (University of Bristol), The Queen's Veterinary School Hospital (University of Cambridge), The Royal (Dick) School of Veterinary Studies Hospital for Small Animals (University of Edinburgh) and Small Animal Department of the Faculty of Veterinary (Ghent University) and 1 private referral practice (Highcroft Veterinary Referrals, CVS group).

To be included in this study, cats needed to have complete medical records, including signalment, general physical and neurological examination findings, recorded presence of urinary or fecal incontinence on presentation, any other concurrent diseases associated with SAD localization, magnetic resonance imaging (MRI) of the affected spinal cord segments and the treatment elected. All imaging studies were reviewed by a specialist in neurology or diagnostic imaging specialist in every institution.

Magnetic resonance imaging machines were all high field (1 machine was 1 Tesla and the others 1.5 Tesla), except for 1 case that low field (0.2 Tesla) was used. Characteristic MRI features included a combination of a tear‐drop or scalpel‐blade‐shaped expansion of the subarachnoid space forming a diverticulum causing secondary spinal cord compression, with a typical appearance being homogenously hyperintense on T2‐weighted (T2W) sequences, isointense to hypointense on T1‐weighted (T1W) sequences, and hypointense on fluid‐attenuated inversion recovery (FLAIR) sequences, when compared to normal spinal cord parenchyma.^1^ In some cases, when available, MR myelogram sequences, half‐Fourier acquisition single‐shot turbo spin‐echo pulse sequence (HASTE), and T2‐weighted thin‐slice gradient echo sequence (BALT GRAD) were used to facilitate visualization of the SAD. The presence of syringomyelia (SM) was also recorded. Syringomyelia was defined as an intramedullary lesion with the same signal intensity as the cerebrospinal fluid (CSF) in the subarachnoid space (hyperintense in T2W and hypointense in T1W, compared to normal spinal cord parenchyma), as previously reported.^14^ The SAD conformation was defined as caudal or cranial direction, depending on the orientation of the “tail” (direction where the CSF dorsal column reduced in a linear way from SAD compression).

Treatment modality was retrieved from medical records and included medical or surgical options. For each case, the decision for treatment was made by the owners based on information about the diagnosis by a specialist in veterinary neurology or a neurology resident in training under the direct supervision of a specialist in veterinary neurology. Medical treatment consisted of a combination of controlled exercise, physiotherapy, analgesia (when necessary), and medications to decrease CSF production, or a combination of these medications. The different surgical techniques used for each case were recorded. Surgeries were performed by a specialist in veterinary neurology or a specialist in veterinary surgery.

The outcome of each case was obtained for short‐ and long‐term periods, if available. For short‐term outcomes, the clinical records of recheck examinations conducted up to 12 weeks following diagnosis were obtained. For long‐term outcomes, information was obtained either from available clinical records of reevaluations or through telephone conversations with referring veterinary practices, from 12 weeks until the last available record. Outcome was classified as improvement, static, or deterioration, based on the reexamination notes, compared to the initial clinical presentation.

Cats were divided into the presence or absence of a concurrent spinal disease associated with the SAD. Statistical analysis was performed using commercially available statistical software (SPSS Statistics for OSx, Version 27, IBM Corp). Age and body weight were tested for normality using Kolmogorov‐Smirnov test. Both were normally distributed, and data were reported as mean and SD (±), and a Student's *T*‐test was used to compare between the 2 groups of cats. The proportion of each sex, breed, treatment option, short‐term, and long‐term outcome were compared between the 2 groups using a Pearson chi‐square test. Statistical significance was defined as *P* < .05.

## 3 RESULTS

**FIGURE 1:**
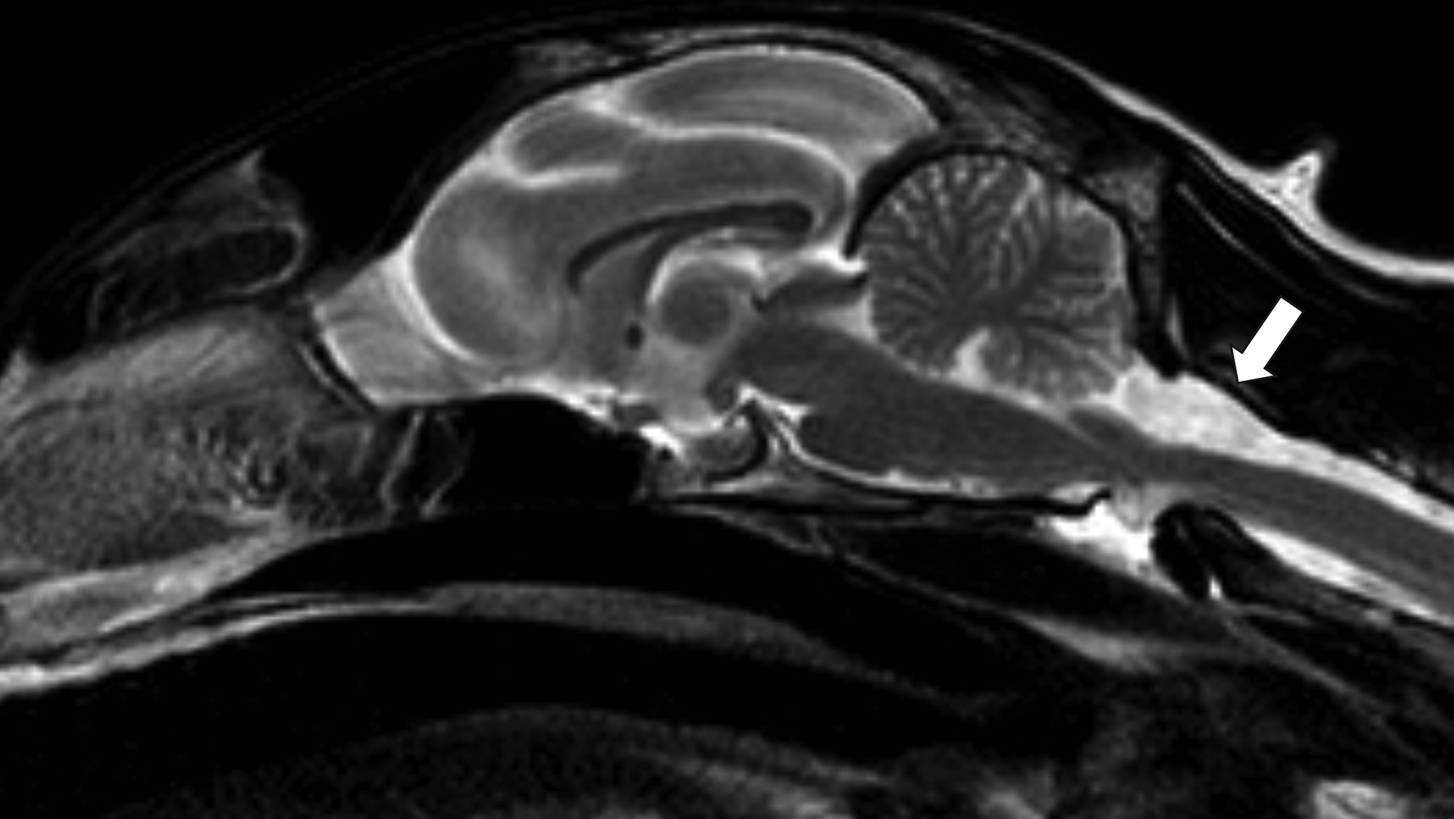
Sagittal T2‐weighted magnetic resonance image of a 7‐year‐old, male neutered, Domestic Short Hair, presented with a 3‐month progressive bilateral symmetrical vestibular ataxia. Presumptive primary spinal arachnoid diverticulum at C1, with a dorsal tear‐drop shape (arrow), and no evidence of syringomyelia.

**FIGURE 2:**
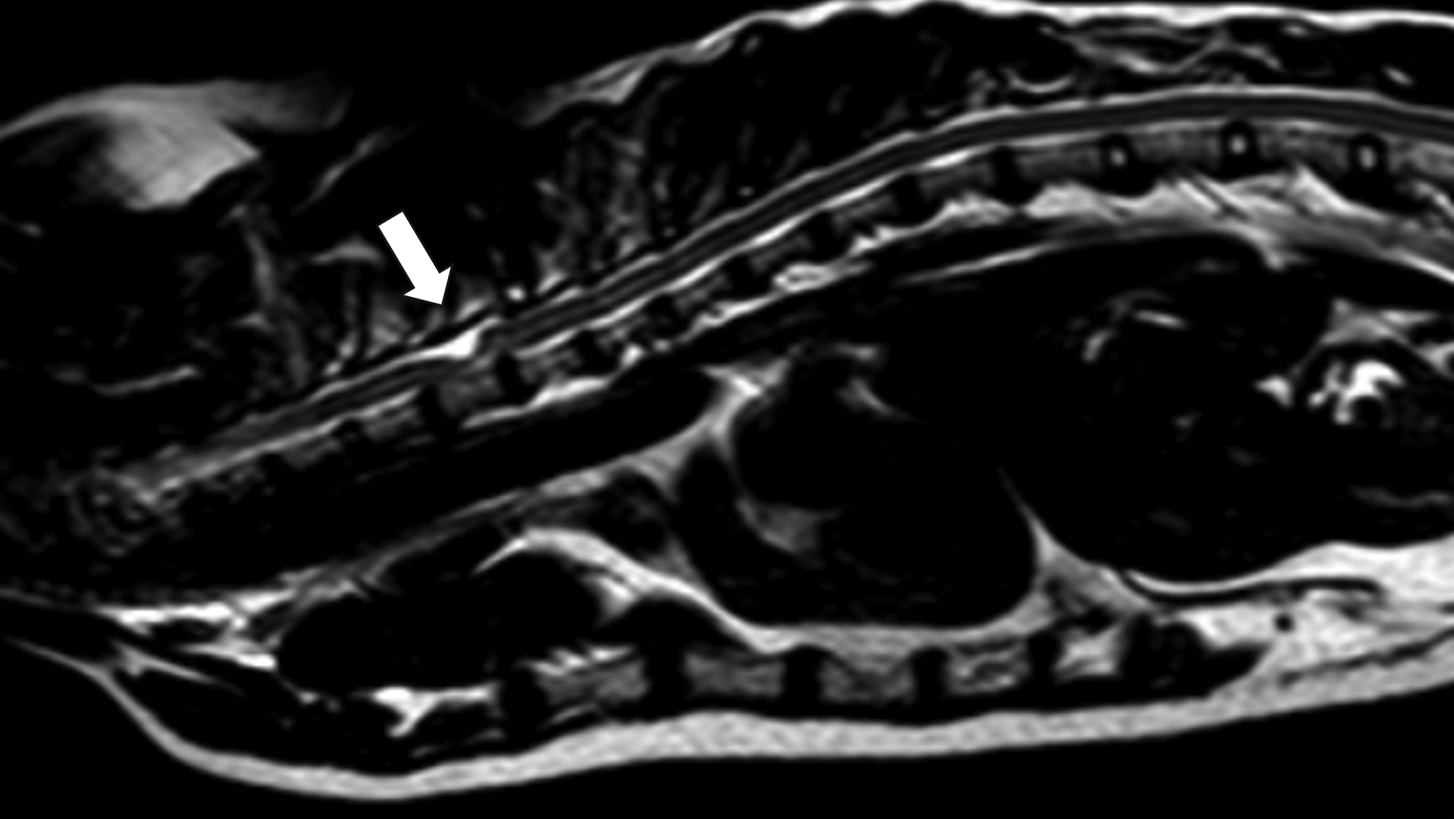
Sagittal T2‐weighted magnetic resonance image of a 12‐year‐old, female neutered, Domestic Short Hair with 24‐months progressive ambulatory paraparesis and fecal incontinence. Presumptive primary spinal arachnoid diverticulum from T3 to T5, with a dorsal tear‐drop shape (arrow), cranial direction, and no evidence of syringomyelia. Although other intervertebral discs displayed degeneration and mild herniation, this was not considered to be clinically relevant.

**FIGURE 3:**
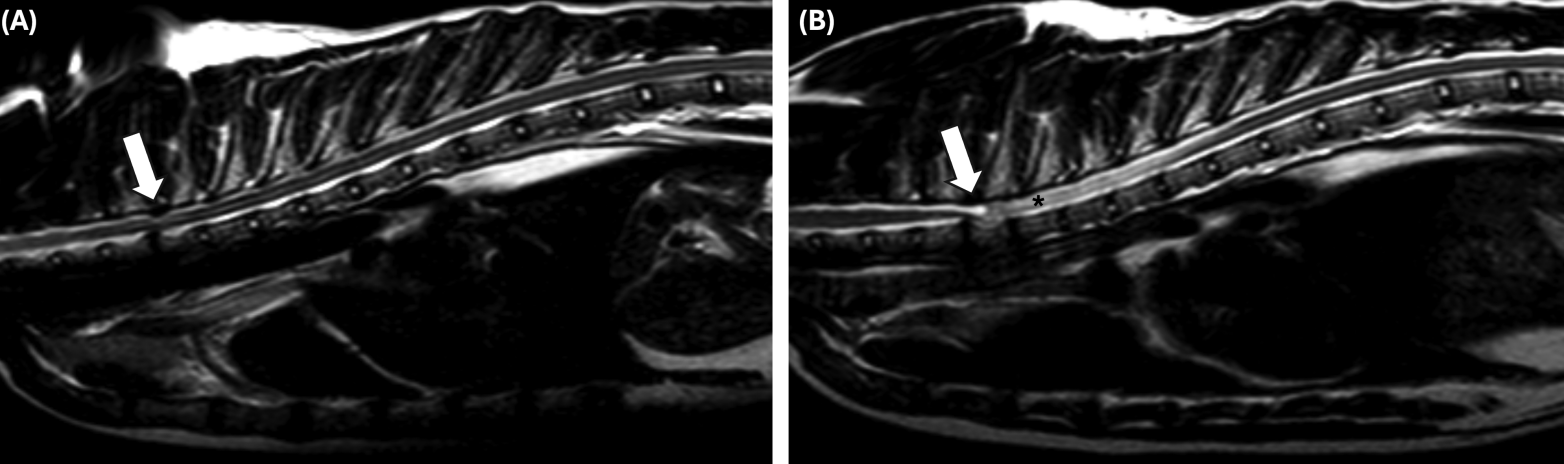
Sagittal T2‐weighted magnetic resonance images of an 8 years old, male neutered, Main Coon with a T2/T3 intervertebral disc protrusion and ligamentum flavum thickening (arrow) that was treated conservatively (A). Same cat, 4 years later, with an acquired spinal arachnoid diverticulum from T2 to T3, with a dorsal tear‐drop shape (arrow), cranial direction, and presence of extensive syringomyelia (asterisk) (B).

**FIGURE 4:**
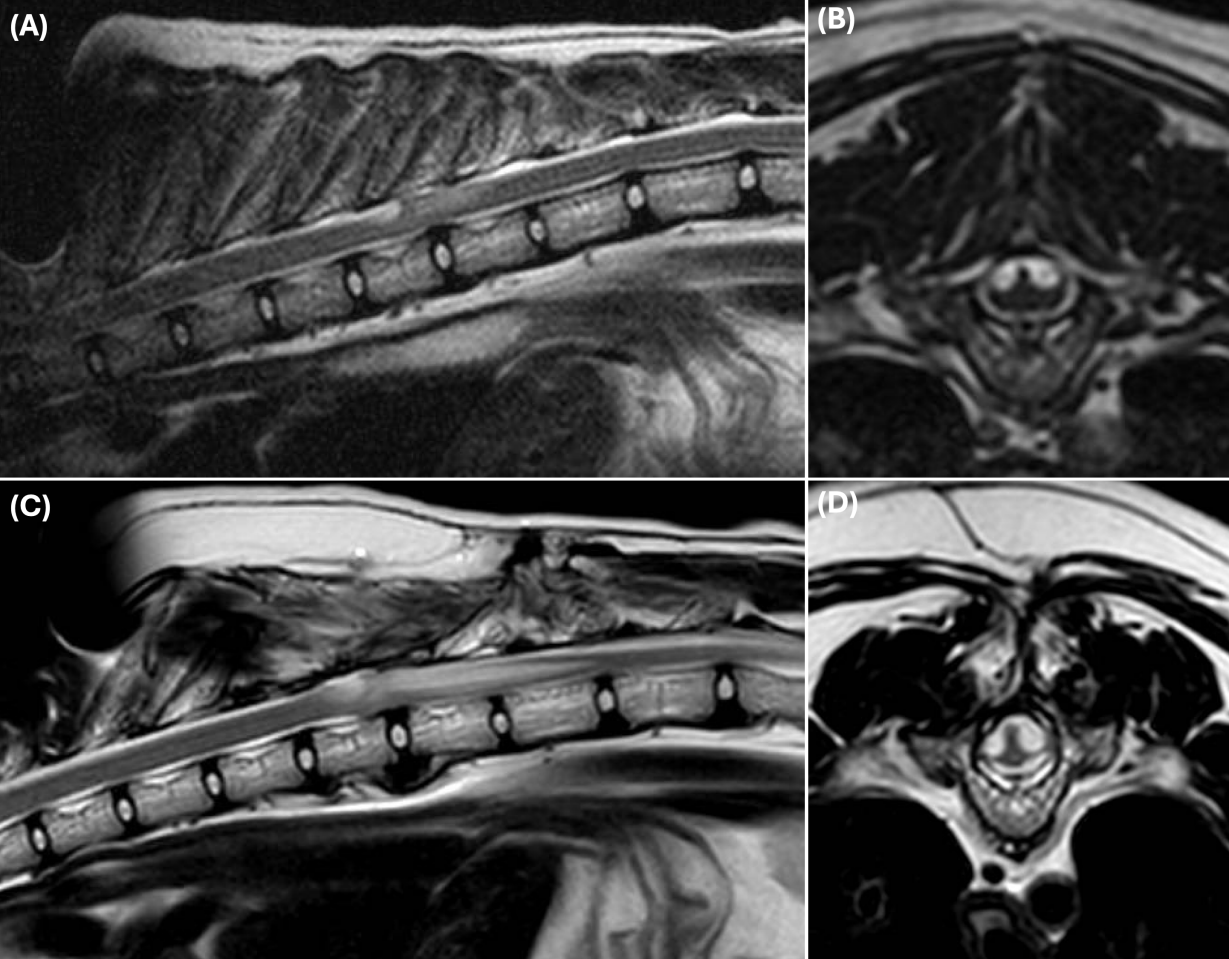
Sagittal and transverse T2‐weighted magnetic resonance images of a 7 years old, male neutered, Ragdoll with a presumptive primary bilobed T9 spinal arachnoid diverticulum (SAD), with a dorsal tear‐drop shape, cranial direction, and evidence of mild syringomyelia (A and B) that was treated with a dorsal laminectomy and durectomy, returning to a normal gait. Sagittal and transverse T2‐weighted magnetic resonance images of the same cat, with a SAD recurrence at the same site, with a similar conformation (C and D). An increased spinal cord compression and extensive syringomyelia can be observed.

A total of 21 cats were included. More detailed information about each cat can be found in Table S1. The most prevalent breed was Domestic Short Hair (14/21; 67%), followed by Domestic Long Hair (3/2; 1 14%), British Short Hair (1/21; 5%), Ragdoll (1/21; 5%), Bengal (1/21; 5%) and Maine Coon (1/21; 5%). There were 8 (38%) females and 13 (62%) males. Median age was 8 years and 4 months (± 3 years and 6 months) and the median body weight was 4.4 kg (± 1.2). All cats were presented with chronic clinical signs, ranging from 1 to 24 months of duration. Most of the cats presented with ambulatory paraparesis and pelvic limb ataxia (17/21; 81%). However, 1 cat demonstrated signs of bilateral symmetrical vestibular and proprioceptive ataxia, 2 were ambulatory tetraparetic and 1 was nonambulatory paraparetic. Spinal hyperesthesia could not be elicited in any of the cats. Most cats (19/21; 90%) had bilateral symmetrical clinical signs, except for 2 cats with more pronounced left‐sided postural reaction deficits, which were not evident during gait assessment. Neuroanatomical localization was consistent with a T3‐L3 myelopathy in 18 (86%) and C1‐C5 myelopathy in 3 (14%) of the 21 cats. The cat with bilateral symmetrical vestibular ataxia had a cervical and brain MRI and was diagnosed with a dorsal C1 SAD, with no abnormalities found in the brain (Figure 1). One of the 21 cats (5%) demonstrated fecal incontinence and was diagnosed with a dorsal T3 to T5 SAD (Figure 2). No cat demonstrated urinary incontinence. Regarding localization of cervical SAD (3/21; 14%), they were found in the cranial cervical region located at the level of C1 (1/3; 33%) and C2 (2/3; 67%). Thoracic SAD localization was found in most cats (14/21; 67%), whereas lumbar SAD was seen in 4 cats (4/21; 19%). Of the 21 cats, 18 (86%) had a bilateral symmetrical dorsal SAD, 1 (5%) had a bilateral asymmetrical (left‐sided) dorsal SAD at the level of T2/T4 and 2 (9%) had a bilateral symmetrical ventral SAD located at the level of L4/L5 and T5. Regarding the SAD conformation, information was available in 19 (91%) of the 21 cats. All cats had a cranial direction SAD conformation, except 1 at the level of C2 which was caudal direction. The presence of SM was identified in 11 (58%) of the 19 cats. In all cases, SM was identified caudally to the SAD. A concurrent disease associated with the SAD localization was found in 11 (52%) of the 21 cats, which included an intervertebral disc herniation in 3 cats (Figure 3), a previous vertebral fracture/luxation in 3 cats, a vertebral malformation in 2 cats, a previous hemilaminectomy site for the treatment of intervertebral disc disease in 2 cats and diagnosis of congenital hypothyroidism that resulted in vertebral epiphysial dysgenesis. The latter has previously been published as a case report.^11^ The only nonambulatory paraparetic cat on presentation was euthanized following diagnosis without treatment. Medical treatment was started in 13 (65%) of 20 cats. Medical treatment consisted of controlled exercise in all cats, prednisolone therapy in 11, and physical rehabilitation in 2 of the 13 cats. One medically treated cat deteriorated and subsequently underwent surgical treatment. A total of 8 (40%) of the 20 cats were treated surgically. Surgical treatment included dorsal laminectomy and durectomy in 6 cats (with 1 having a titanium mesh applied over the laminectomy site) and hemilaminectomy and durotomy in 2 cats. The outcome was available for 19 of the 21 cats (95%). Of the 2 cats with missing outcome information, 1 was euthanized following diagnosis, and the other, which received medical treatment, was lost to follow‐up. For the short‐term outcome of 20 cases (including the cat's outcome after both medical treatment and subsequent surgery), 6 (30%) deteriorated, 9 (45%) improved and 5 (25%) remained static, with a time frame of follow‐up information ranging from 2 to 12 weeks. Long‐term outcome was available in 11 (52%) of the 21 cats. For the 11 cats long‐term outcome, 8 (73%) deteriorated, 2 (18%) improved and 1 (9%) remained static, with a time frame of follow‐up information ranging from 11 to 36 months. In the short‐term outcome for the 8 surgically managed cats, 6 (75%) improved, 1 (12.5%) deteriorated and 1 (12.5%) remained static compared with the 12 medically managed cats where 3 (25%) improved, 5 (42%) deteriorated and 4 (33%) remained static. In the long‐term for the 6 surgically managed cats, 2 (40%) improved whereas 4 (60%) deteriorated, compared with the 5 medically managed cats where 4 (80%) cats deteriorated (only 1 showed a transient initial improvement) and 1 (20%) cat that remained static. Only 2 cats, that were previously treated with surgery, underwent repeat MRIs confirming a recurrence of SAD (Figure 4) on the same site 14 and 21 months after the first diagnosis. The cat with 14‐month SAD recurrence was previously included in another study.^12^

When comparing cats with concurrent diseases associated with SAD to those without, there was no statistical difference in age (mean = 104 vs 86 months; *P* = .337), body weight (mean = 4.7 vs 3.9 kg; *P* = .165), breed (*P* = .15), sex (*P* = .864), type of treatment (*P* = .284), short‐term outcome (*P* = .447), and long‐term outcome (*P* = .484).

## 4 DISCUSSION

This study describes the clinical presentation, diagnostic imaging findings, treatment, and outcome in a group of cats with SAD. In contrast to published reports^4^, ^5^, ^6^, ^7^, ^8^, ^9^, ^10^, ^11^ where all cases of SAD in cats were associated with underlying previous or concurrent spinal conditions, in this study, there was no lesion found in association with SAD in 48% of the cats. Although it is possible that our diagnostic investigations failed to identify subtle or previously healed concurrent conditions, this finding might represent primary SAD in cats. Although primary SAD is typically assumed to be congenital, the clinical signs in presumptive primary SAD cases in this study developed later in life. The underlying etiology of cats with suspected primary SAD remains uncertain. No difference in age, body weight, breed, gender, treatment, or outcome was observed between cats with suspected primary or acquired (secondary to a spinal disease) SAD.

Regarding clinical features, there were more male cats (62%) than females (38%) with a diagnosis of SAD. Intriguingly, the same has been described in dogs.^1^, ^15^, ^16^ In humans, this prevalence remains unclear.^17^, ^18^ A wide range of ages was described in this study, from 18 weeks to 13 years old. This could represent the heterogenous character of concurrent spinal conditions and the unclear etiology of suspected primary SAD in cats. As for the clinical presentation, most of the cats presented with a typical presentation for chronic and progressive myelopathy. One cat presented with signs of bilateral vestibular and proprioceptive ataxia with a dorsal SAD to C1 spinal cord segment. Vestibular clinical signs related to a cervical spinal cord lesion have been described and it is suspected to be related to the involvement of the cervicospinovestibular tract which has muscle proprioceptors from spinal nerve, dorsal roots or dorsal gray matter of the C1/C3 spinal cord segments, that are part of the vestibular proprioceptive system.^19^, ^20^ None of the cats were painful and no marked lateralization of the clinical signs was observed in most of the cases (90%). This could potentially be explained by the slow progressive nature of this condition. This is also in agreement with findings in dogs with the same condition.^1^, ^21^ The assessment of spinal hyperesthesia in cats is considered subjective because it relies on the individual's interpretation of a cat's reaction to spinal palpation, which can be inaccurate.^22^, ^23^, ^24^ Different pain assessment scores have been validated for feline patients, such as the feline musculoskeletal pain index (FMPI) or the feline grimace scale (FGS).^23^, ^24^ However, the retrospective nature of this study precluded the use of these pain assessment scores. Another possible explanation for these clinical features could be related to the SAD localization in the vertebral canal. In this cohort, the SAD was located dorsally in most of the cats (n = 19/21; 90%) and there was a moderate prevalence of SM associated with SAD localization (n = 11/19; 58%). Moreover, SAD was found to cause a bilateral symmetrical compression of the spinal cord in most cases (20/21; 95%). However, a cautious interpretation of the relationship of these imaging features with the clinical signs is warranted. It remains challenging to conclude whether syringomyelia is present secondary to the SAD or sequelae of a previously resolved spinal condition.

Only 1 cat (5%), with a dorsal T3/T5 SAD, suffered fecal incontinence at the time of presentation. However, 1 cat developed urinary and fecal incontinence and 1 cat developed urinary incontinence during long‐term follow‐up. These cats had initially improved after surgery with subsequent deterioration and confirmed recurrence of SAD on the same localization. Fecal and urinary incontinence, or a combination of these, is reported in dogs with SAD, with a prevalence of around 8%, and is primarily associated with thoracolumbar SAD.^1^, ^2^, ^3^, ^13^ This is also reported in humans with spinal arachnoid intradural cysts, with a wider prevalence ranging from 7% to 50%.^25^, ^26^ It is hypothesized that urinary and fecal incontinence, or a combination of these, is caused by a sphincter dysfunction secondary to disruption of the dorsally located ascending sensory pathways.^1^, ^3^, ^13^, ^27^

Six (75%) cats improved after surgery, whereas 3 (24%) cats improved after medical management in the short‐term outcome. Although this finding could suggest that surgery is associated with a better short‐term outcome, results could have been affected by the lack of investigation of factors influencing the decision to treat medically and surgically, which was not possible given the retrospective nature of the study. Comparison of medical vs surgical treatment was not performed given the retrospective nature of this study, low number of cases (insufficient power), and the heterogeneous character of the included cats. However, 73% (8/11) of the cats deteriorated, regardless of treatment, on available long‐term follow‐up. This could be related to concurrent spinal conditions affecting the treatment success, but no obvious association was found in this study.

Limitations of this study include its retrospective and multicenter nature, which meant that treatment protocols were not standardized, and information collected might have been incomplete. Furthermore, the MRI machines were different between centers which could have influenced the ability to accurately describe SAD conformation and the presence of secondary SM. The presence of SM in cats has been reported in 2 case reports.^4^, ^10^ Furthermore, cases that were classified as primary SAD did not have computed tomography to rule out inconspicuous vertebral malformations, such as caudal articular process dysplasia. Long‐term follow‐up information was obtained in approximately half of the cats, which might have influenced the results for the long‐term outcome and recurrences could have been missed. Moreover, long‐term information was mainly obtained by telephone interviews, which is a subjective assessment of clinical progression.

Although the results of this study confirmed that most cats with SAD have a potential underlying spinal condition, in almost half of the cats in this study no underlying spinal condition could be identified. Nevertheless, no differences in signalment, treatment, and outcome between suspected primary and acquired SAD were identified. Only 1 cat was presented with fecal incontinence in this cohort of cats. Most of the cats could be treated successfully with surgery for the short‐term outcome. However, for the long‐term outcome, a large proportion of the cats (73%) deteriorated, regardless of the therapy elected. Although SAD in cats is considered a rare disease, this condition should be included in the list of differential diagnoses for cats presenting with chronic, progressive, nonlateralized, nonpainful myelopathy, particularly if they are male and have a history of spinal disease or surgery.

## CONFLICT OF INTEREST DECLARATION

Authors declare no conflict of interest.

## OFF‐LABEL ANTIMICROBIAL DECLARATION

Authors declare no off‐label use of antimicrobials.

## INSTITUTIONAL ANIMAL CARE AND USE COMMITTEE (IACUC) OR OTHER APPROVAL DECLARATION

Approved by the Social Sciences Research Ethical Review Board (SSRERB) at the Royal Veterinary College (RVC), reference number URN SR2021‐0149.

## HUMAN ETHICS APPROVAL DECLARATION

Authors declare human ethics approval was not needed for this study.

## Supporting information


**Table S1.** Table with information collected for the 21 cats including breed, age, sex, presence of incontinence, previous or concurrent spinal conditions, spinal arachnoid diverticulum (SAD) localization, treatment modality, and summary outcome. BSH, British Short Hair; DLH, Domestic Long Hair; DSH, Domestic Short Hair; FE, female entire; FN, female neutered; IVDE, intervertebral disc extrusion; IVDP, intervertebral disc protrusion; m, months; ME, male entire; MN, male neutered; RTA, road traffic accident; w, weeks; y, years.
