# Supplementary material for: Spinal arachnoid diverticula in cats: Clinical presentation, diagnostic imaging findings, treatment, and outcome
Source: J Vet Intern Med. 2024 Dec 30;39(1):e17294. doi: 10.1111/jvim.17294 (PMC11683399; doi:10.1111/jvim.17294)
Supplement: Supplementary file 1 — Table S1. Table with information collected for the 21 cats including breed, age, sex, presence of incontinence, previous or concurrent spinal conditions, spinal arachnoid diverticulum (SAD) localization, treatment modality, and summary outcome. BSH, British Short Hair; DLH, Domestic Long Hair; DSH, Domestic Short Hair; FE, female entire; FN, female neutered; IVDE, intervertebral disc extrusion; IVDP, intervertebral disc protrusion; m, months; ME, male entire; MN, male neutered; RTA, road traffic accident; w, weeks; y, years. [file JVIM-39-e17294-s001.docx]

**Table 1 -** Table with information collected for the 21 cats including breed, age, sex, presence of incontinence, previous or concurrent spinal conditions, spinal arachnoid diverticulum (SAD) localisation, treatment modality and summary outcome. DSH: Domestic Short-Hair; DLH: Domestic Long-Hair; BSH: British Short-Hair; y: years; m: months; w: weeks; MN: male neutered; ME: male entire; FN: female neutered; FE: female entire; IVDE: intervertebral disc extrusion; IVDP: intervertebral disc protrusion; RTA: road traffic accident.

| **Cat** | **Breed** | **Age** | **Sex** | **Incontinence** | **Previous or concurrent spinal conditions** | **SAD localization** | **Treatment** | **Outcome** |
| --- | --- | --- | --- | --- | --- | --- | --- | --- |
| 1 | DLH | 18w | MN | No | None | Dorsal L2 | Surgical - Dorsal laminectomy and durectomy | Initial deterioration and static pre-operative state 6w later. |
| 2 | DLH | 5y | FS | No | None | Dorsal T9/T10 | Medical - Prednisolone | Recovered to normal gait. Mild episodes of pelvic limb ataxia over a period of 4y. |
| 3 | DSH | 12y7m | FS | No | T9/T10 IVDP | Dorsal T9 | Medical - Prednisolone | No change after 5w. |
| 4 | DSH | 10y8m | FE | No | None | Dorsal T12/L1 | Surgical - Dorsal laminectomy and durectomy | Improvement after 4w. |
| 5 | DLH | 7y | ME | No | None | Dorsal T10/L1 | Medical -Physiotherapy | Static for 1y |
| 6 | DSH | 13y | FE | No | T10/T11 IVDE - right sided hemilaminectomy 5y prior to presentation | Dorsal T10/T11 | Medical - Prednisolone | Static for 3m. |
| 7 | Ragdoll | 7y | ME | No | None | Dorsal T9 | Surgical - Dorsal laminectomy and durectomy | Return to normal. Recurrence of clinical signs 14m later confirmed with MRI with SAD on the same site. Second surgery performed (extended dorsal laminectomy to T7). Improvement following surgery but remained with pelvic limb ataxia. Developed urinary incontinence 33m later. Euthanasia 43m later following acute paraplegia with negative nociception, without MRI to confirm diagnosis. |
| 8 | DSH | 11y9m | ME | No | T3/T4 vertebral malformation | Dorsal T2/T4 | Medical - Prednisolone | Lost to follow up |
| 9 | DSH | 3y1m | ME | No | L3/L4 luxation following an RTA 2 years prior to presentation | Dorsal L3/L4 | Surgical - Dorsal laminectomy and durectomy | Improvement. Recurrence of clinical signs 21m later, with urinary and faecal incontinence. Confirmed SAD relapse at same site with MRI and medical treatment started (gabapentin, meloxicam and physiotherapy). |
| 10 | BSH | 6y6m | FE | No | None | Dorsal C2 | Medical - Prednisolone | Initial improvements and deteriorated with mild left pelvic limb monoparesis 28m later. |
| 11 | DSH | 9y | FE | No | Lumbosacral subluxation following RTA 5 years prior to presentation | Ventral L4/L5 | Medical - Physiotherapy | Deteriorated 4w later and euthanatized. |
| 12 | DSH | 12y | FE | Faecal incontinence | None | Dorsal T3/T5 | Surgical - Left sided hemilaminectomy and durotomy | Static following surgery. Acutely deteriorated after 2m. |
| 13 | DSH | 7y | ME | No | None | Ventral T5 | Medical - Prednisolone | Deteriorated 4w later and euthanatized. |
| 14 | Bengal | 8y | ME | No | T3/T4 vertebral malformation | Dorsal T3 | None | Euthanasia |
| 15 | DSH | 7y | ME | No | None | Dorsal C1 | Surgical - Dorsal laminectomy and durectomy | Initial improvement. Slow deterioration on 11m re-check. |
| 16 | DSH | 11y | ME | No | No | Dorsal T7 | Medical - Prednisolone | Deteriorated and euthanasia 11m later. |
| 17 | DSH | 11y | MN | No | T10/T11 IVDP | Dorsal T10/T11 | Medical - Prednisolone | Improvement after 3w |
| 18 | DSH | 9y | MN | No | L2/L3 luxation following an RTA 2 years prior to presentation | Dorsal L3/L4 | Medical - Prednisolone | Slowly deteriorated and euthanasia 24m later. |
| 19 | Maine Coon | 8y | MN | No | T2/T3 IVDP | Dorsal T2/T3 | Medical - Prednisolone | Deteriorated and euthanasia 4w. later. |
| 20 | DSH | 9y1m | FN | No | T3/T4 IVDP and ligament flavum hyperplasia and hemilaminectomy for removal of extradural material | Dorsal T3/T4 | Medical – Prednisolone  Surgery -  Durectomy and titanium mesh | No improvement with medical treatment for 21 days.  Initial improvement after surgery followed by deterioration and euthanasia. |
| 21 | DSH | 1y4m | MN | No | Congenital hypothyroidism | Dorsal C2 | Surgical – Hemilaminectomy and durotomy | Neurologically normal 9m later. |
